# Effects of an alternative host on the prevalence and intensity of infection of a bumble bee parasite

**DOI:** 10.1017/S003118202200004X

**Published:** 2022-04

**Authors:** Mario S. Pinilla-Gallego, Rebecca E. Irwin

**Affiliations:** Department of Applied Ecology, North Carolina State University, Raleigh, NC 27695, USA

**Keywords:** *Bombus impatiens*, *Crithidia bombi*, serial passage

## Abstract

Several bee parasites are transmitted through flowers, and some of them can infect multiple host species. Given the shared use of flowers by bee species, parasites can potentially encounter multiple host species, which could affect the evolution of parasite virulence. We used the trypanosomatid parasite *Crithidia bombi* and its host, the common eastern bumble bee (*Bombus impatiens*), to explore the effect of infecting an alternative host, the alfalfa leaf-cutter bee (*Megachile rotundata*), on parasite infectivity and ability to replicate. We conducted a serial passage experiment on primary and alternative hosts, assessing infectivity and intensity of infection during five passes. Parasite cells from each pass through the alternative host were also used to infect a group of primary hosts. We found that serial passes through the alternative host increased infectivity, but there was no effect on intensity of infection. Interestingly, both the probability and intensity of infection on the primary host increased after serial passage through the alternative host. This increase in intensity of infection could be due to maladaptation after selection of new *C. bombi* strains has occurred in the alternative host. This study suggests that host switching has the potential to affect the adaptation of bee parasites to their hosts.

## Graphical abstract

**Figure:**
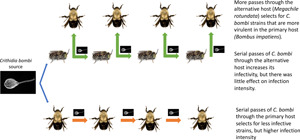

## Introduction

Most parasites have multiple host species, and hosts are usually attacked by multiple parasite species, with important ecological and evolutionary implications for both hosts and parasites (Rigaud *et al*., 2010). These multi-species interactions make zoonotic diseases and emerging infectious diseases (EIDs) a threat not only for wildlife, but also for humans and domestic animals (Daszak *et al*., 2000), highlighting the importance of understanding and being able to predict the evolutionary path of parasites (Betts *et al*., 2016). One important factor is the evolution of virulence. Theoretical studies suggest that in a scenario where host species differ in quality for a parasite, parasite populations should evolve towards optimal virulence in their primary host, and suboptimal virulence in alternative host species (Gandon, 2004). However, these predictions have rarely been tested in field or laboratory experiments, making it difficult to predict the evolutionary trajectory of parasites, which has implications for the management of human and wildlife EIDs (Rigaud *et al*., 2010).

Bees (Hymenoptera: Apoidea) host a wide variety of parasites (Shimanuki and Knox, 2000; Goulson and Hughes, 2015), several of which can infect host species in different genera or even families (e.g. Ngor *et al*., 2020), and have been linked to the population decline of managed and wild bees (VanEngelsdorp and Meixner, 2010; Bianco *et al*., 2014). A common route of horizontal transmission of bee parasites is by the shared use of flower resources, as infected individuals deposit parasites on flowers that can be picked up by other bees (Durrer and Schmid-Hempel, 1994; Graystock *et al*., 2015; Alger *et al*., 2019). Given that both managed and wild bees congregate on patches of flowers (Becker *et al*., 2015; Piot *et al*., 2019) where 10–30% of flowers can have at least one bee parasite (Graystock *et al*., 2020; Piot *et al*., 2020), it is expected that parasites commonly encounter several host species. Variation in host size and immune level can affect the amount of parasite propagules produced by a host (Baer and Schmid-Hempel, 2003; Brown *et al*., 2003*b*; Sinpoo *et al*., 2018), and this heterogeneity in host quality, as well as different encounter rates with different host species, could drive the evolution of virulence of parasites (Gandon, 2004; Wilber *et al*., 2020).

Due to the threat of EIDs, it is important to understand the processes that drive virulence evolution of parasites in multi-host communities (Wilber *et al*., 2020), and understand the role that biodiversity plays in the host–parasite interaction. Here, we use the parasite *Crithidia bombi* (Trypanosomatida: Trypanosomatidae) and its host, the common eastern bumble bee *Bombus impatiens* (Hymenoptera: Apidae), to explore the effect of an alternative host on parasite virulence and infectivity. Specifically, we ask whether infecting an alternative host and the number of passes through the alternative host influence: (1) the parasite's ability to infect the primary and alternative host, and (2) the intensity of infection in the primary and alternative host. We predicted that both the ability to infect a host and intensity of infection would increase in a particular host after serial passes in that host, as the parasite would presumably become more adapted to that host. We also predicted that infectivity and intensity of infection in the primary host would be suboptimal after serial passage of the parasite through the alternative host, assuming that the parasite becomes more adapted to the alternative host (Yañez *et al*., 2020).

## Materials and methods

### Study system

*Crithidia bombi* is an intestinal parasite of bumble bees (*Bombus* spp.) that reproduces in the hindgut lumen with new cells released to the environment in bee feces (Schmid-Hempel and Schmid-Hempel, 1993; Imhoof and Schmid-Hempel, 1999). Although it can be a benign parasite when the host is under optimal conditions (Brown *et al*., 2000; Yourth and Schmid-Hempel, 2006), *C. bombi* can reduce the number of new queens produced in wild bumble bee colonies (Goulson *et al*., 2018) and the success rate of infected overwintering queens when starting a nest in the spring (Schmid-Hempel, 2001). *Crithidia bombi* reproduces clonally, but there can be genetic exchange, and new strains are produced in 7–16% of infections (Schmid-Hempel *et al*., 2011; Tognazzo *et al*., 2012). This genetic variability is also reflected in strong genotype–genotype interactions between the parasite and its host (Schmid-Hempel *et al*., 1999; Barribeau *et al*., 2014; Marxer *et al*., 2016).

As a host we used *B. impatiens*, which is a eusocial bee species native to eastern North America, and is commercially reared for agricultural pollination (Kleijn *et al*., 2015). As a generalist forager, *B. impatiens* visits a variety of plant species that span a diversity of floral traits (Mader *et al*., 2010).

Recently, Ngor *et al*. (2020) found that *C. bombi* can infect and actively replicate in the alfalfa leaf-cutter bee (ALCB) *Megachile rotundata* (Apidae: Megachilidae), a commercial solitary bee originally from Europe, that is now widely distributed in North America (Mader *et al*., 2010). Adults emerge in late-spring and fly for a period of approximately 1 month. In cooler climates, pre-pupae will enter diapause and complete development the following spring, but in warmer climates, pupae can complete development the same year and have a second generation that will overwinter until the following spring (Pitts-Singer and Cane, 2011). Given the overlapping flying periods of *B. impatiens* and *M. rotundata* during summer months and their ability to visit similar flowers (Scott-Dupree *et al*., 2009; Mader *et al*., 2010), there is the potential for the sharing of parasites on flowers between these two species. Hereafter, *B. impatiens* is referred to as the primary host, and ALCB as the alternative host.

### Bee sources and maintenance

#### Bumble bees

Commercial colonies of *B. impatiens* were obtained from Koppert Biological Systems (Howell, MI, USA), maintained in a dark room at approximately 27°C and 50% RH, and provided sugar water (30% sucrose) and honey bee collected pollen (CC High Desert Pollen, Phoenix, AZ, USA) *ad libitum*. Upon arrival, all colonies were screened for *C. bombi* infection. We always maintained two to three uninfected colonies as a source of experimental bees, and one to two colonies infected with *C. bombi* isolated from *B. impatiens* collected at Stone Soup Farm, Hadley, MA (GPS coordinates: 42.363911 N, −72.567747 W) as sources of *C. bombi*.

#### Alfalfa leaf-cutter bees

ALCB cocoons were obtained from JWM Leafcutters, Inc. (Nampa, ID, USA). Cocoons were stored at 4°C in a plastic container with a mesh lid, and then incubated at 30°C until emergence (approximately 3 weeks). Once bees emerged, they were transferred to a small container with access to sucrose solution *ad libitum*. Bees used in the experiments were 1–3 days old.

### Inoculum preparation

#### From source colonies

To prepare inoculum to start each replicate, a standard protocol was followed (Richardson *et al*., 2015). Briefly, 8–10 workers were collected from a *C. bombi*-infected bumble bee colony. Guts of each individual bee were dissected and homogenized separately in 300 *μ*L distilled water (dH_2_O). Samples were allowed to settle for 3–4 h to allow the gut debris to sink to the bottom of the tube. Two-hundred microlitres of clean supernatant from each sample were taken and mixed together, and a 10 *μ*L aliquot was used to estimate the number of *C. bombi* cells per microlitre in a Neubauer chamber with a compound microscope at 400× magnification. Then, dH_2_O and 50% sucrose were used to dilute the mixture to 25% sucrose with 1200 cells *μ*L^−1^.

#### From experimental bees

To prepare inoculum from experimental bees, guts of each individual bee were dissected. In the case of bumble bees, each gut was homogenized in 300 *μ*L dH_2_O; in the case of ALCBs, each gut was homogenized in 50 *μ*L dH_2_O, as these bees are much smaller than bumble bees (intertegular distance 2.3–2.8 mm for ALCBs compared to 3.6–3.9 mm for *B. impatiens*; Adhikari *et al*., 2019). Samples were allowed to settle for 3–4 h, and then a 10 *μ*L aliquot was used to estimate the number of *C. bombi* cells in the sample, as mentioned above. We mixed the supernatant of samples with positive counts within each species, and used a new sample to determine the *C. bombi* concentration. Then, equal parts of the guts and 50% sucrose were mixed to make an inoculum that was 25% sucrose. Because the number of infected bees and the level of infection of experimental bees was variable, the concentration of the inoculum prepared from experimental bees was variable each time it was prepared. We controlled for this variability in the statistical analysis described below. Inoculum from ALCBs was prepared with guts from 7–23 bees, and it ranged from 275 to 1200 cells *μ*L^−1^ with a mean of 820 cells *μ*L^−1^ (Table S1). Inoculum from experimental bumble bees was prepared with guts from 2–7 bees and it ranged from 25 to 1200 cells *μ*L^−1^ with a mean of 680 cells *μ*L^−1^ (Table S1).

The right forewing from each experimental bee was collected to measure the length of the radial cell as an estimate of bee size (bumble bees: Müller *et al*., 1996; ALCBs: Appendix S1), using ImageJ software (V 1.8). Bee size was not used in the statistical models described below, however, because size covaried with species (*B. impatiens* being dramatically larger than ALCBs).

### Experimental set-up

**Fig. 1.**
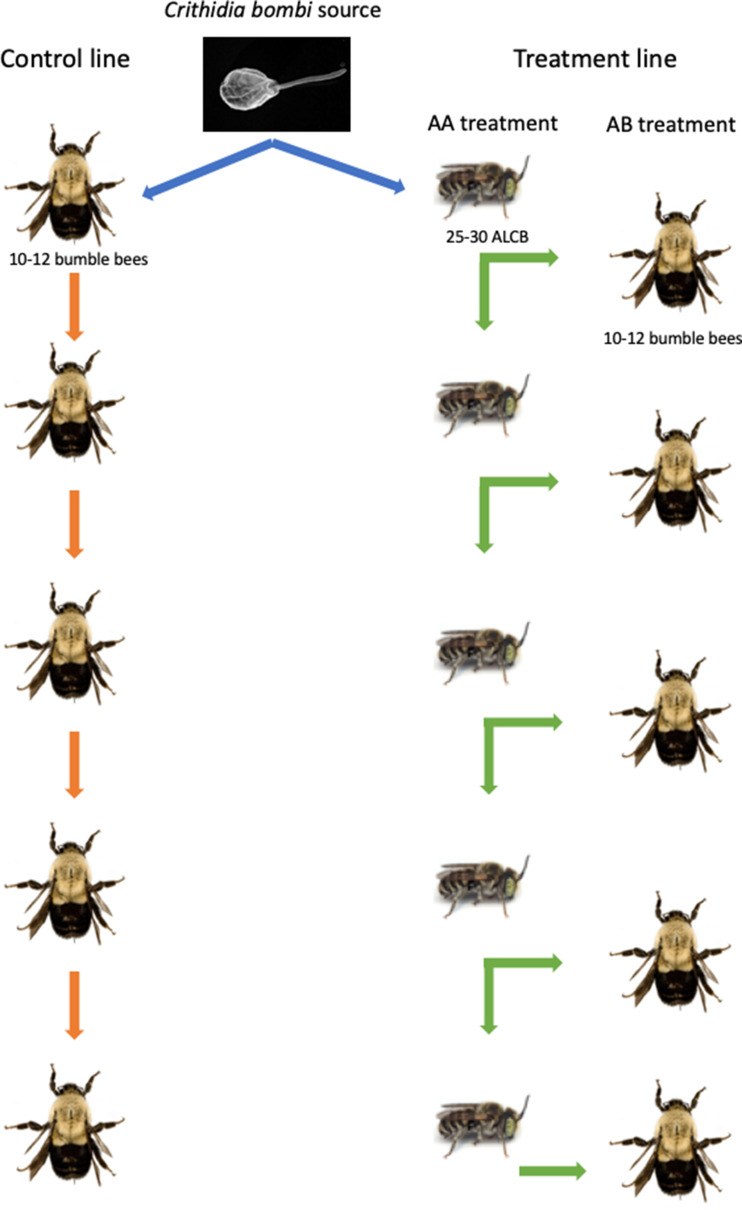
Experimental design. In this SPE, *Crithidia bombi* from a source colony was used to infect a group of bumble bees (control line) and ALCBs (AA treatment). One week after infection, guts were dissected and this *C. bombi* was used to inoculate the next group of bees. In the case of ALCBs, part of the inoculum was also used to infect a group of bumble bees (AB treatment).

To test the effect of infecting an alternative host on *C. bombi* infectivity and intensity of infection, we set-up a serial passage experiment (SPE) (Fig. 1). To start each replicate, inoculum was prepared from a bumble bee source colony (see section ‘Inoculum preparation’), and used to inoculate 10–12 bumble bees (control) and 25–35 ALCBs (treatment). Each bumble bee received 10 *μ*L inoculum whereas each ALCB received 5 *μ*L, again given that ALCBs are smaller than bumble bees. Each individual bee was maintained in 15 mL plastic vials (6.1 mm *h* × 3.3 mm *d*) with sucrose and pollen *ad libitum*, refreshed every other day. After 7 days, the parasites attained a representative level in bumble bees (Logan *et al*., 2005) and so we dissected each individual bumble bee and ALCB and determined whether they were infected and the intensity of infection (as in section ‘Inoculum preparation’). Guts from bumble bee controls were used to make inoculum for another passage through bumble bees, simulating a situation in which the parasite transmits between primary hosts only. Guts from ALCBs were used to make inoculum for another passage through ALCBs (AA treatment). To test the effect of infecting an alternative host on the ability of the parasite to infect the primary host, *C. bombi* from ALCBs were used to inoculate a group of 10–12 bumble bees (AB treatment, Fig. 1) that were dissected 1 week after inoculation. We had six replicates; one replicate had two passages, two replicates had three passages, one replicate had four passages and two replicates had five passages. The variable number of passages in each replicate was due to the availability of newly emerged ALCBs on the inoculation date.

### Statistical analysis

All data analyses were performed using R (v. 4.02) (R Core Team, 2018). ‘Incidence’ (presence/absence of *C. bombi* infection) and ‘intensity of infection’ (*C. bombi* counts from infected bees) were analysed using generalized linear mixed models (GLMMs).

#### Incidence analysis

We modelled parasite incidence using logistic regression with the package glmmTMB (Skaug *et al*., 2018). The response variable was the binary outcome of whether a bee was infected or not. The full model included treatment (control, AA and AB treatments; see Fig. 1), the number of passages, an interaction term between those two factors and the inoculum concentration used to inoculate each group of bees. Bee species was included as a random effect. To determine the significance of the fixed effects, a likelihood ratio test comparing the full model with a model that excluded each of the fixed effects as an explanatory variable was conducted. Non-significant terms were removed, and the model with the lowest Akaike information criterion value was selected. Model assumptions were evaluated by generating QQ plots of residual *vs* predicted with the DHARMa package (Hartig and Lohse, 2020). To explore the effect of interactions, we calculated the odds ratio and conducted pairwise comparisons of the slope of each treatment with the ‘emtrends’ function of the emmeans package (Russell *et al*., 2021).

#### Intensity of infection analysis

To model intensity of *C. bombi* infection, only data from infected bees (positive counts) were used (Table S1). The cell count per 0.02 *μ*L gut sample was used as the response variable. These data were highly right-skewed, so log-transformation was used, followed by a GLMM with Gaussian distribution. The same fixed and random effect terms as in the incidence analysis were used. Model selection, test of model assumptions and exploration of interactions of the models were performed in the same way as in the incidence analysis.

## Results

### Incidence of infection

**Fig. 2.**
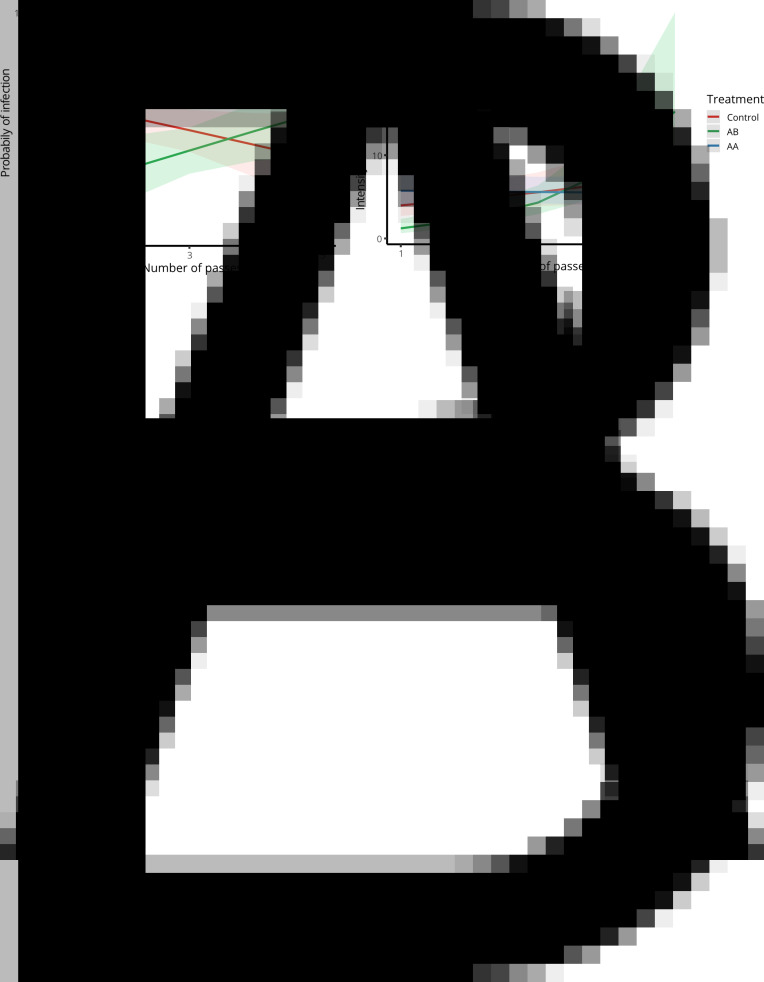
(A) Predicted incidence of infection and (B) predicted intensity of infection (cells per 0.02 *μ*L) for: control, *Bombus impatiens* to *B. impatiens*, AA, *Megachile rotundata* to *M. rotundata* and AB, *M. rotundata* to *B. impatiens*.

Treatment was a significant predictor of the probability of infection (χ^2^_2_ = 31.64, *P* < 0.0001), whereas the number of passes was not (χ^2^_1_ = 2.56, *P* = 0.109). However, there was a significant interaction between the treatment and the number of passes (χ^2^_2_ = 8.98, *P* < 0.011; Fig. 2A). Although each additional pass through the bumble bee control group reduced the probability of infection by 20%, each pass through the alternative host (AA treatment) and back to the primary host (AB treatment) increased the probability of infection by 20 and 32%, respectively (Fig. 2A; Table S2). Pairwise comparisons revealed that while there was no difference between the slopes of the AA and AB treatments (*t*_927_ = 0.55, *P* = 0.84), both treatments were significantly different from the control (*t*_927_ = −2.75, *P* = 0.017; *t*_927_ = −2.44, *P* = 0.039, respectively). The inoculum concentration was also a significant predictor in the model, with higher concentration increasing the probability of infection (χ^2^_1_ = 24.27, *P* < 0.0001).

### Intensity of infection

The number of passes was a significant predictor (χ^2^_2_ = 6.23, *P* = 0.013), whereas treatment was not a significant predictor for the intensity of the infection (χ^2^_1_ = 0.58, *P* = 0.75). But, similar to the incidence analysis, there was a significant interaction between the treatment and the number of passes (χ^2^_2_ = 23.97, *P* < 0.0001; Fig. 2B). Although each additional pass from bumble bee to bumble bee (control) and from an alternative host to alternative host (AA treatment) had little to no effect on the intensity of infection (18% increase and 1% decrease, respectively), each pass through the alternative host increased the intensity of infection on the primary host (AB treatment) by 88% (Fig. 2B; Table S3). The pairwise comparisons confirmed that the slope of the AB treatment was significantly different from that of the control and AA treatment (*t*_619_ = −3.17, *P* = 0.0046; *t*_619_ = 4.53, *P* < 0.001, respectively), but there was no difference between the slopes of the control and AA treatment (*t*_619_ = 1.38, *P* = 0.35). The inoculum concentration was also a significant predictor in the model, with higher concentration increasing the intensity of infection (χ^2^_1_ = 8.31, *P* = 0.003).

## Discussion

SPEs usually find that parasites increase virulence on alternative hosts as selection for transmission is removed and within-host competition selects for faster growing strains that are more adapted to the alternative host (Alizon *et al*., 2013). At the same time, those new strains usually have a suboptimal virulence on other host genotypes (Ebert, 1998). Here, we found that the serial passage of *C. bombi* through the primary bumble bee host selected for less infective strains, but those strains produced more intense infections (higher cell count). Although we expected an increase in the probability of infection, other studies have also found results that deviate from the expectation of SPEs. For example, serial passes of *C. bombi* through bumble bees of the same colony did not increase the intensity of infection (Yourth and Schmid-Hempel, 2006), and acute bee paralysis virus decreased virulence on honey bees after multiple serial passes (Bailey and Gibbs, 1964). Beyond bees and their parasites, Huang *et al*. (2019) found that the fungus *Fusarium oxysporum* experienced decreased virulence after serial passes on susceptible cucumber cultivars. These studies, combined with our results, suggest that an increase in infectivity and intensity of infection is not always the rule in SPEs and that evolution does not always follow simple theoretical expectations (Yourth and Schmid-Hempel, 2006). The mechanism(s) driving the decrease in the probability of infection with serial passage of *C. bombi* through bumble bees is unknown. However, one relevant hypothesis is that constitutive defenses of bumble bee workers, which are part of the immune response to *C. bombi* (Brown *et al*., 2003*a*; Whitehorn *et al*., 2011), increase with colony age (Moret and Schmid-Hempel, 2009), and because we used workers from the same colony for each replicate, workers in later passes could have been more resistant to *C. bombi*. This hypothesis warrants further investigation.

Serial passage of *C. bombi* through the alternative host, *M. rotundata*, increased the probability of infection as expected in an SPE, but it had little effect on the intensity of infection. It is possible that *C. bombi* needs longer exposure to the alternative host before there is a detectable increase in the intensity of infection, as we would expect a lower baseline adaptation to this host. This could be because the rate at which virulence increases in SPEs is slower for eukaryotes compared to viruses and bacteria (Ebert, 1998).

Interestingly, the probability and intensity of infection on the primary bumble bee host decreased after the first pass through the alternative host. But, as the number of passes through the alternative host increased, the probability and intensity of infection on the primary host also increased. It is possible that during the first pass through the alternative host there is strong selection for strains that are able to infect the alternative host, but at the same time have a lower ability to infect bumble bees. The increase in infectivity and intensity of infection on bumble bees after several passes through the alternative host could be due to maladaptation to bumble bees of the strains that are being selected in ALCBs (Gandon, 2004), as higher intensity of infection in bumble bees could reduce survival of the host to the point of decreasing between-host transmission (Leggett *et al*., 2013). A genetic comparison of *C. bombi* strains after serial passes through the primary and alternative hosts could help elucidate any potential genetic changes occurring, and also help us to identify genes that are involved in the evolution of virulence (Gisder *et al*., 2018).

Biodiversity can play an important role in host–parasite dynamics, and it has been argued that increasing host community diversity could reduce parasite transmission and virulence due to an ‘encounter reduction’ effect (Keesing *et al*., 2006; Johnson *et al*., 2009). This makes it more difficult for parasites to evolve an optimal virulence level for any particular host (Leggett *et al*., 2013), and is something that has been observed in bee communities (Fearon and Tibbetts, 2021). Given that the infectivity and intensity of infection of *C. bombi* on bumble bees after the first pass through the alternative host were lower relative to the control treatment, we would expect that having ALCBs as an alternative host of *C. bombi* in a bee community could decrease its transmission to bumble bees, as the new strains are less infective and less virulent. Additionally, ALCBs are smaller than bumble bees and should produce fewer new parasite cells, decreasing the amount of propagule in the environment. Although multiple passes through ALCBs eventually increased infectivity and intensity of infection on bumble bees relative to the control treatment, we consider that the chances of *C. bombi* to be transmitted multiple times through ALCBs under natural conditions are low, and therefore we may not expect these highly virulent strains to appear in the wild.

Testing the predictions of mathematical models for the evolution of virulence of multi-host parasites is essential to manage EIDs of humans and wildlife (Perlman and Jaenike, 2003), for example, by identifying host maintenance potential in multi-host parasite communities (Wilber *et al*., 2020). Parasites and diseases are considered one of the main factors contributing to decline in bee populations (Goulson *et al*., 2008; Potts *et al*., 2010), so understanding the factors that affect parasite virulence could lead to the development of strategies to mitigate these declines. Our study is subject to the limitations of a laboratory SPE, but our results suggest that having *M. rotundata* as an alternative host in bee communities could slow down the spread of *C. bombi* to bumble bees, supporting the idea that higher biodiversity can counterbalance the spread of parasites. Future studies should explore more realistic scenarios, including incorporating the effects of transmission on flowers rather than serial passages, multiple host species, multiple infections and the natural phenology of primary and alternative hosts.

## Data Availability

Data and code from this study are available from the Dryad Digital Repository: https://doi.org/10.5061/dryad.02v6wwq53.

## Supplementary material

For supplementary material accompanying this paper visit https://doi.org/10.1017/S003118202200004X. click here to view supplementary material
