# Supplementary material for: Effects of an alternative host on the prevalence and intensity of infection of a bumble bee parasite
Source: Parasitology. 2022 Jan 24;149(4):562–7. doi: 10.1017/S003118202200004X (PMC10090601; doi:10.1017/S003118202200004X)
Supplement: Supplementary file 1 [file S003118202200004Xsup001.docx]

SUPPLEMENTARY MATERIAL: Effects of an alternative host on the virulence of a bumble bee parasite


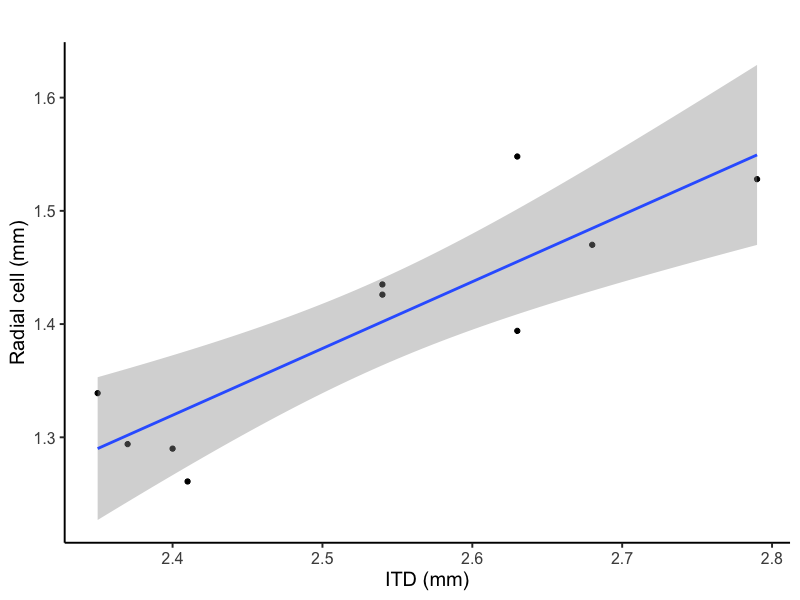


Figure S1: Relationship between length of the radial cell and intertegular distance (ITD) of *Megachile rotundata*.

Table S1: Summary information for each passage. Numbers represent Mean ± S.E. “Bees used for inoculum” is the number of individual bees we took guts from to make inoculum for that passage. “Bees used in the trial” is the number of bees we inoculated for each passage. “Bees that became infected” is the number of bees that had positive cell counts and therefore were used for the analysis of the intensity of infection.

| Passes | Treatment | Bees used for inoculum | Inoculum concentration (cell/μl) | Bees used in the trial | Bees that became infected |
| --- | --- | --- | --- | --- | --- |
| Pass 1 | Control | 6 ± 0.5 | 1300 ± 63 | 10 ± 1 | 9 ± 1 |
|  | AA | 6 ± 0.5 | 1300 ± 63 | 29 ± 2 | 22 ± 3 |
| Pass 2 | Control | 5 ± 0.6 | 783 ± 199 | 11 ± 0.3 | 6 ± 1 |
|  | AA | 17 ± 2.5 | 533 ± 164 | 24 ± 3 | 17 ± 2 |
|  | AB | 19 ± 2.0 | 600 ± 168 | 10 ± 0.2 | 5 ± 1 |
| Pass 3 | Control | 3 ± 0.7 | 385 ± 165 | 10 ± 1 | 3 ± 1 |
|  | AA | 13 ± 3.0 | 681 ± 205 | 26 ± 6 | 20 ± 4 |
|  | AB | 13 ± 3.0 | 681 ± 205 | 9 ± 1 | 4 ± 1 |
| Pass 4 | Control | 3 ± 1.5 | 392 ± 183 | 9 ± 1 | 5 ± 3 |
|  | AA | 17 ± 2.5 | 505 ± 246 | 28 ± 1 | 17 ± 1 |
|  | AB | 17 ± 2.5 | 500 ± 251 | 10 ± 0.3 | 4 ± 2 |
| Pass 5 | Control | 3 ± 0.5 | 1038 ± 113 | 6 ± 2 | 5 ± 4 |
|  | AA | 15 ± 5.5 | 375 ± 25 | 22 ± 7 | 19 ± 5 |
|  | AB | 15 ± 5.5 | 375 ± 25 | 10 ± 1 | 8 ± 2 |

Table S2: Odds ratio of the incidence model.

| *Predictors* | *Odds Ratios* | *CI* | *p* |
| --- | --- | --- | --- |
| (Intercept) | 1.37 | 0.60 – 3.12 | 0.451 |
| treatment [AB] | 0.16 | 0.05 – 0.58 | 0.005 |
| treatment [AA] | 0.66 | 0.29 – 1.48 | 0.314 |
| Passes | 0.80 | 0.62 – 1.04 | 0.096 |
| Inoculum concentration | 1.00 | 1.00 – 1.00 | <0.001 |
| treatment [AB] * passes | 1.66 | 1.11 – 2.50 | 0.015 |
| treatment [AA] * passes | 1.50 | 1.12 – 2.01 | 0.006 |

Table S3: Odds ratio of the intensity model.

| *Predictors* | *Odds Ratio* | *CI* | *P* |
| --- | --- | --- | --- |
| (Intercept) | 2.35 | 1.28 -4.26 | 0.005 |
| passes | 1.18 | 1.00 -1.39 | 0.049 |
| treatment [AB] | 0.19 | 0.08 -0.48 | <0.001 |
| treatment [AA] | 0.72 | 1.00 – 2.94 | 0.05 |
| Inoculum concentration | 1 | 1.00 -1.00 | 0.004 |
| passes * treatment [AB] | 1.60 | 1.20 - 2.14 | 0.002 |
| passes * treatment [AA] | 0.84 | 0.69 - 1.01 | 0.064 |
